# 

**Published:** 1897-12

**Authors:** 


### BOOKS RECEIVED.

Lippincott's Pocket Medical Directory, including the pronunciation and definition of 20,000 of the principal terms used in medicine and the allied sciences, together with many elaborate tables. Edited by Ryland W. Greene, A. B., Editor of Lippincott's Medical Dictionary. Philadelphia and London: J. B. Lippincott & Co. 1897.

Index Catalogue of the Library of the Surgeon-General's Office, United States Army. Authors and Subjects. Second series. Vol. II. B. Bywater. Washington: Government Printing Office. 1897.

A Treatise on Gynecology, Medical and Surgical. By Dr. S. Pozzi, of Paris. Third revised edition. Translated by Dr. Brooks H. Wells, of New York. One volume, of 950 pages, royal octavo, illustrated by over 600 wood engravings. Muslin, $5.50 net; leather, $6.50 net. New York: William Wood & Company. 1897.

A Text-Book of Practical Therapeutics, with especial reference to the application of Remedial Measures to Disease and their Employment upon a Rational Basis. By Hobart Amory Hare, M. D., Professor of Therapeutics and Materia Medica in the Jefferson Medical College, Philadelphia, etc. With special chapters by Drs. George E. de Schweinitz, Edward Martin and Barton C. Hirst. Sixth edition, thoroughly revised and largely rewritten. In one octavo volume of 756 pages. Cloth, $3.75; leather, $4.75. Philadelphia and New York: Lea Brothers & Co., Publishers. 1897.

International Clinics. A Quarterly of Clinical Lectures on Medicine, Neurology, Surgery, Gynecology, Obstetrics, Ophthalmology, Laryngology, Pharyngology, Rhinology, Otology and Dermatology, and specially prepared articles on treatment. By professors and lecturers in the leading medical colleges of the United States, Germany, Austria, France, Great Britain and Canada. Edited by Judson Daland, M. D., (Univ. of Penna.) Philadelphia, Instructor in Clinical Medicine and Lecturer on Physical Diagnosis in the University of Pennsylvania; Assistant Physician to the Hospital of the University of Pennsylvania, etc.; Fellow of the College of Physicians of Philadelphia. J. Mitchell Bruce, M. D., F. R. C. P., London, England, Physician to, and Lecturer on, the Principles and Practice of Medicine at the Charing Cross Hospital. David W. Finlay, M. D., F. R. C. P., Aberdeen, Scotland, Professor of Practice of Medicine in the University of Aberdeen; Physician to, and Lecturer on, Clinical Medicine in the Aberdeen Royal Infirmary, etc. Volume III. Seventh series. 1897. Octavo, pp. xii.—360. Philadelphia: J. B. Lippincott Co. 1897.

A Practical Treatise on Diseases of the Skin. By John V. Shoemaker, M. D., LL. D., Professor of Skin and Venereal Diseases in the Medico-Chirurgical College and Hospital of Philadelphia; Physician to the Philadelphia Hospital for Diseases of the Skin, etc., etc. Octavo, pp. xiii.—894. Third edition, revised and enlarged, with chromogravure plates and other illustrations. New York: D. Appleton & Company. 1897.

Skin Diseases of Children. By George Henry Fox, A. M., M. D., Clinical Professor of Diseases of the Skin, College of Physicians and Surgeons, New York; Physician to the New York Skin and Cancer Hospital, etc. Octavo, pp. viii.—166. With twelve photogravure and chromographic plates and sixty illustrations in the text. New York: William Wood & Company. 1897.

A Handbook of Therapeutics. By Sidney Ringer, M. D., F. R. S., Holme Professor of Clinical Medicine, University College; Physician to University College Hospital, and Harrington Sainsbury, M. D., F. R. C. P., Physician to the Royal Free Hospital, and the City of London Hospital for Diseases of the Chest, Victoria Park. Thirteenth edition. Octavo, pp. xi.—746. New York: William Wood and Company. 1897.

Handbook of Materia Medica, Pharmacy and Therapeutics, including the physiological action of drugs, the special therapeutics of disease, official and practical pharmacy and minute directions for prescription writing. By Samuel O. L. Potter, A. M., M. D., M. R. C. P., London, Professor of the Principles and Practice of Medicine and Clinical Medicine in the College of Physicians and Surgeons of San Francisco; Medical Superintendent of St. Mark's Hospital, etc. Sixth edition. Fully revised and greatly enlarged. Royal octavo, pp. xv.—900. Price, cloth, $4.50 net; leather, $5.50 net. Philadelphia: P. Blakiston, Son & Company, 1012 Walnut street. 1897.

A Text-book of the Diseases of Women. By Henry J. Garrigues, A. M., M. D., Professor of Gynecology in the New York School of Clinical Medicine; Gynecologist to St. Mark's Hospital in New York City, etc. Octavo, pp. 728. Containing 335 engravings and colored plates. Second edition, thoroughly revised. Price, cloth, $4.00 net; half morocco, $5.00 net. Philadelphia: W. B. Saunders, 925 Walnut street. 1897.

Clinical Diagnosis: The Bacteriological, Chemical and Microscopical Evidence of Disease. By Dr. Rudolf v. Jaksch, Professor of Special Pathology and Therapeutics, and Director of the Medical Clinic in the German University of Prague. Translated from the fourth German edition and enlarged by James Cagney, M. A., M. D., Member of the Royal College of Physicians of London; Physician to the Hospital for Epilepsy and Paralysis, etc. Third edition. Octavo, pp. xxv.—523. With numerous illustrations (partly in colors). Price, $6.50. London: Charles Griffin & Company, Limited; Exeter street, Strand. 1897.

Transactions of the American Surgical Association. Volume XV. Edited by DeForest Willard, A. M., M. D., Ph. D., Recorder of the Association. Printed for the Association. For sale by William J. Dornan, Philadelphia. 1897.

Manual of Gynecology. By Henry T. Byford, Professor of Gynecology and Clinical Gynecology in the College of Physicians and Surgeons of Chicago; Professor of Gynecology and Clinical Gynecology in the Woman's Medical School of Northwestern University; Professor of Gynecology in the Post-Graduate Medical School of Chicago. Octavo, pp. xxiii—596, containing 341 illustrations, many of which are original. Price, $3.00. Philadelphia: P. Blakiston, Son & Company, 1012 Walnut street. 1897.

Simon's Clinical Diagnosis. New 2d) edition, revised and enlarged. A manual of clinical diagnosis by microscopical and chemical methods. For students, hospital physicians and practitioners. By Charles E. Simon, M. D., late Assistant Resident Physician Johns Hopkins Hospital, Baltimore. In one very handsome octavo volume of 530 pages, with 135 engravings and 14 full-page colored plates. Cloth, $3.55. Philadelphia and New York: Lea Brothers & Co. 1897.

Medical Education and Registration. United States and Canada. By William T. Slayton, M. D. (Harv.), Hyde Park, Vermont. Published annually. Price, 75 cents. 1897.

Text-Book of Nervous Diseases. By Charles L. Dana, A. M., M.D., New York. Fourth revised edition. One volume, octavo, 640 pages. Profusely illustrated. Muslin, $3.50 net. New York: William Wood & Company. 1897.

Twenty-third Annual Report of the Secretary of the State Board of Health of the State of Michigan for the fiscal year ending June 30, 1895. Lansing: Robert Smith Printing Co. 1896.

Therapeutics, its Principle and Practice. By H. C. Wood, M. D., LL. D., Professor of Materia Medica and Therapeutics, and Clinical Professors of Diseases of the Nervous System in the University of Pennsylvania. A work on medical agencies, drugs and poisons, with especial reference to the relations between physiology and clinical medicine. Tenth edition, thoroughly revised. Octavo, pp. xxxi.—1033. Philadelphia: J. B. Lippincott Co. 1897.

Abbott's Bacteriology. Fourth and revised edition. Just ready. The Principles of Bacteriology. A practical manual for students and physicians. By A. C. Abbott, M. D., Professor of Hygiene and Director of the Laboratory of Hygiene, University of Pennsylvania, Philadelphia. Fourth edition, enlarged and thoroughly revised. Handsome 12mo, 542 pages, 106 illustrations, of which 19 are colored. Cloth, $2.75. Philadelphia and New York: Lea Brothers & Co., Publishers

An Epitomé of the History of Medicine. By Roswell Park, A. M., M. D., Professor of Surgery in the Medical Department of the University of Buffalo, etc. Illustrated with portraits and other engravings. One volume, royal octavo, pages xiv.—348. Extra cloth, beveled edges, $2.00 net. Philadelphia: The F. A. Davis Co., Publishers, 1914 and 1916 Cherry street; New York: 117 W. Forty-second street; Chicago: 9 Lakeside Building.

Practical Diagnosis. The Use of Symptoms in the Diagnosis of Disease. By Hobart Amory Hare, M. D., Professor of Therapeutics and Materia Medica in the Jefferson Medical College of Philadelphia, Laureate of the Medical Society of London, of the Royal Academy in Belgium, etc. New (2d) and revised edition. In one octavo volume of 598 pages, with 201 engravings and 13 full-page colored plates. Cloth, $4.75. Philadelphia: Lea Brothers & Co., Publishers. 1897.

Vade Mecum of Ophthalmological Therapeutics. By Dr. Landolt and Dr. Gygax. Price, $1.00. Philadelphia: J. B. Lippincott Co. 1898.

